# Performance of Plant-Growth-Promoting Rhizobacteria (PGPR) Isolated from Sandy Soil on Growth of Tomato (*Solanum lycopersicum* L.)

**DOI:** 10.3390/plants12081588

**Published:** 2023-04-09

**Authors:** Medhat Rehan, Ahmad Al-Turki, Adil H. A. Abdelmageed, Noha M. Abdelhameid, Ayman F. Omar

**Affiliations:** 1 Department of Plant Production and Protection, College of Agriculture and Veterinary Medicine, Qassim University, Buraydah 51452, Saudi Arabia; trky@qu.edu.sa (A.A.-T.); ad.abdelmageed@qu.edu.sa (A.H.A.A.); a.mohmed@qu.edu.sa (A.F.O.); 2 Department of Genetics, Faculty of Agriculture, Kafrelsheikh University, Kafr El-Sheikh 33516, Egypt; 3 Department of Horticulture, University of Khartoum, Khartoum North, Shambat 13314, Sudan; 4 Soil Fertility and Microbiology Department, Desert Research Center (DRC), Cairo 11753, Egypt; nmousa5@gmail.com; 5 Plant Pathology and Biotechnology Lab, EPCRS Excellence Center, Department of Plant Pathology, Faculty of Agriculture, Kafrelsheikh University, Kafr El-Sheikh 33516, Egypt

**Keywords:** PGPR, sustainability, IAA, P solubilization, food security, environmental protection, tomato plant

## Abstract

The plant-growth-promoting rhizobacteria (PGPR) in the rhizosphere affect plant growth, health, and productivity, as well as soil-nutrient contents. They are considered a green and eco-friendly technology that will reduce chemical-fertilizer usage, thereby reducing production costs and protecting the environment. Out of 58 bacterial strains isolated in Qassim, Saudi Arabia, four strains were identified by the 16S rRNA as the *Streptomyces cinereoruber* strain P6-4, *Priestia megaterium* strain P12, *Rossellomorea aquimaris* strain P22-2, and *Pseudomonas plecoglossicida* strain P24. The plant-growth-promoting (PGP) features of the identified bacteria involving inorganic phosphate (P) solubilization, the production of indole acetic acid (IAA), and siderophore secretion were assessed in vitro. Regarding the P solubilization, the previous strains’ efficacy reached 37.71%, 52.84%, 94.31%, and 64.20%, respectively. The strains produced considerable amounts of IAA (69.82, 251.70, 236.57, and 101.94 µg/mL) after 4 days of incubation at 30 °C. Furthermore, the rates of siderophore production reached 35.51, 26.37, 26.37, and 23.84 psu, respectively, in the same strains. The application of the selected strains in the presence of rock phosphate (RP) with tomato plants under greenhouse conditions was evaluated. The plant growth and P-uptake traits positively and significantly increased in response to all the bacterial treatments, except for some traits, such as plant height, number of leaves, and leaf DM at 21 DAT, compared to the negative control (rock phosphate, T2). Notably, the *P. megaterium* strain P12 (T4), followed by *R. aquimaris* strain P22-2 (T5), revealed the best values related to plant height (at 45 DAT), number of leaves per plant (at 45 DAT), root length, leaf area, leaf-P uptake, stem P uptake, and total plant P uptake compared to the rock phosphate. The first two components of the PCA (principal component analysis) represented 71.99% (PCA1 = 50.81% and PCA2 = 21.18%) of the variation at 45 DAT. Finally, the PGPR improved the vegetative-growth traits of the tomato plants through P solubilization, IAA, and siderophore production, and ameliorated the availability of nutrients. Thus, applying in PGPR in sustainable agriculture will potentially reduce production costs and protect the environment from contamination by chemical fertilizers and pesticides.

## 1. Introduction

In the rhizosphere area, plant-growth-promoting rhizobacteria (PGPR) are present and enhance plant growth by secreting a wide range of metabolites and enzymes [1]. These PGPR have many mechanisms to support plant growth, such as phytohormone production (i.e., cytokinins, auxin, gibberellins, abscisic acid), phosphate and potassium solubilization, siderophore secretion for iron uptake, 1-Aminocyclopropane-1-carboxylate deaminase (ACC) production, nitrogen fixation, antifungal activities, volatile organic compound (VOC) production, and the enhancement of plant–microbe symbiosis [2,3,4,5]. Plant-growth-promoting rhizobacteria GPR are considered attractive and environmental-friendly replacements for chemical fertilizers and pesticides. These microorganisms represent a potentially advantageous technique for enhancing crop productivity, quality, and security for sustainable agriculture. Furthermore, plant growth-promoting microbes (PGPMs) secrete large quantities of substances that indirectly influence overall plant morphology [3,6].

The influence of PGPR on plant growth is due to the production of auxin phytohormones. Many bacterial strains can produce indolic compounds, such as indole-3-acetic acid (IAA), which increases the levels of auxin in plants, and subsequently improves plant growth by enhancing water and nutrient uptake, increases cell division, differentiates vascular tissues, initiates the formation of lateral and adventitious roots, and improves resistance to abiotic stresses [1,6,7,8,9,10]. Additionally, phosphorus (P) is considered an essential element for plants and its limited bioavailability in soil affects plant growth; thus, inorganic phosphate solubilization and mineralization by PGPR is an important trait. Furthermore, the solubilization of inorganic phosphate can occur as a result of the secretion of some organic acids, such as citric and gluconic acids. Otherwise, the mineralization process may occur through the synthesis of different phosphatases that catalyze phosphoric-ester hydrolysis [8,11,12,13].

Iron is an important microelement, and it is necessary for chlorophyll biosynthesis, photosynthesis process and respiration. By producing siderophores (low-molecular-weight molecules, 400–2000 Da), microorganisms chelate iron with high affinity and deposit it within their cells. In this way, siderophores improve plant nutrition (direct effect) and control phytopathogen growth (indirect effect) via the chelation of iron from the surrounding environment [6,8,14,15].

The tomato (*S. lycoperiscum* L.) is the second most important vegetable crop in the world [16], and its production is heavily concentrated in semi-arid regions [17]. In addition to its economic importance, the tomato is an ideal material for physiological, cellular, biochemical, and molecular-genetic investigations. In terms of human health, the tomato is a major component of daily meals in many countries and constitutes an important source of minerals, vitamins, and antioxidant compounds [18]. In epidemiological studies, tomato fruit had one of the highest inverse correlations with cancer risk and cardiovascular diseases, including stroke [16]. The versatility of the tomato fruit contributes greatly to its popularity as a food product. It is one of the main greenhouse-vegetable crops and is widely grown in Saudi Arabia. The total greenhouse area for tomato production in 2020 was 12,454.3 hectares, producing 351,212.4 tons [19,20].

Chemical fertilizers are frequently used to improve soil fertility and supply plants with the nutrients they need to produce more productive crops. Moreover, the widespread use of these chemical fertilizers pollutes the environment and soil with hazardous substances (such as nitrates) [21,22,23]. Recently, organic agriculture has attracted more attention to conserve the environment and public health. There are many advantages of adding plant-growth-promoting rhizobacteria to soils. They can secrete phytohormones and siderophores, fix nitrogen, solubilize phosphate and potassium, biocontrol phytopathogens, and enhance tolerance of biotic and abiotic stresses [23,24].

Abdelmageed et al. [25], Walpola and Yoon [25], Ahirwar et al. [26], and Puia et al. [27] reported positive and significant effects of biofertilizers on aspects of the vegetative growth (such as shoot length, shoot dry weight, shoot fresh weight, fresh and dry weights of the root, root length), the yield, and the stability of the root-exploration area of tomato plants. The present study aimed to: (1) Isolate new plant-growth-promoting bacteria (PGPB) from the rhizosphere area; (2) identify the selected isolates at the molecular level; (3) evaluate their plant-growth-promoting characteristics in vitro; (4) apply the promising PGPB in vivo with tomato plants under greenhouse conditions; and (5) assess the associations among the evaluated traits.

## 2. Results

### 2.1. Evaluation of the Bacterial Plant-Growth-Promoting Characterstics In Vitro

**Figure 1:**
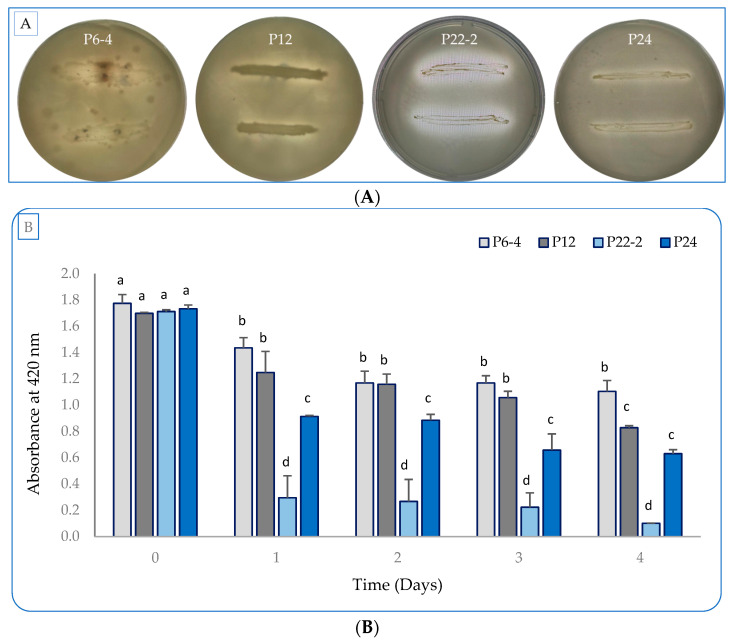
Inorganic-phosphate solubilization by the four isolated bacterial strains (P6-4, P12, P22-2, and P24) grown in NBRIP medium for 4 days. (**A**) The four strains growing on NBRIP agar medium with clear zones. (**B**) The solubilization efficiency of selected strains in NBRIP liquid medium. Each value represents the mean of three replicates. Bars with different letters are significantly different (*p* > 0.05). Error bars represent the standard deviation of the mean.

Out of isolated 58 isolates on the National Botanical Research Institute’s phosphate (NBRIP) agar medium (the developed medium for screening phosphate-solubilizing microorganisms), four designated bacterial isolates (P6-4, P12, P22-2, and P24) were chosen for further study. The selected isolates displayed patterns of phosphate solubilization that were better than those of the other isolates, since they formed clearer zones around the growing cells. The aforementioned strains were evaluated for their efficiency in solubilizing phosphate and producing both IAA and siderophores. The application of the NBRIP agar medium inoculated with the four selected isolates led to phosphate mineralization through the formation of a clear zone around the growing colonies (Figure 1A). To evaluate the solubilization efficiency, the NBRIP broth medium was inoculated with any of the previous isolates for 4 days (Figure 1B). The P-solubilization activity in strain P6-4 reached 37.71% on day 4, whereas P12 exhibited higher activity, with a simultaneous efficiency of 52.84%. Additionally, strain P22-2 had the highest effectiveness of inorganic P bioavailability, with an efficacy measured at 94.31%, followed by strain P24, with 64.20%.

**Figure 2:**
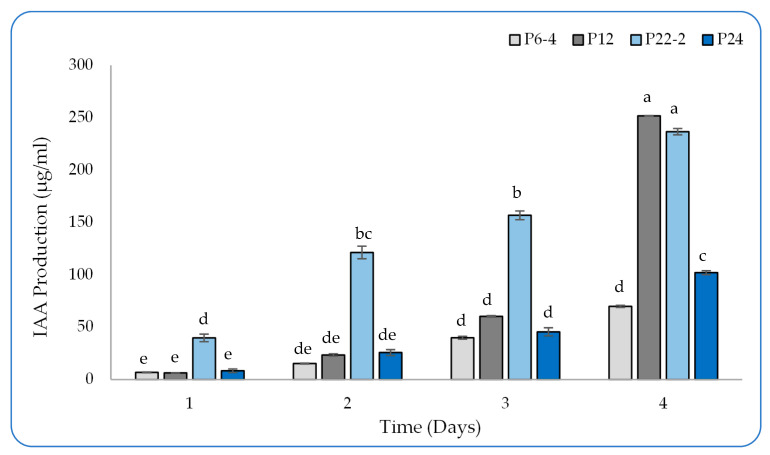
IAA production of the four selected bacterial strains (P6-4, P12, P22-2, and P24) grown in nutrient broth supported by 0.2% L-tryptophan over four days; all values represent the average of three replicates. Bars with different letters are significantly different (*p* > 0.05). Error bars represent the standard deviation of the mean.

The tested strains generated varying amounts of IAA based on their growth in the NB medium supported by L-tryptophane (Figure 2). After 4 days of incubation, the P6-4 produced up to 69.82 µg/mL, while the P24 displayed a higher level of production, reaching 101.94 µg/mL. When incubated for the same period, the highest production assigned to both the P12 and the P22-2 isolate gradually increased in terms of IAA production, reaching values of 251.70 and 236.57 µg/mL, respectively.

**Figure 3:**
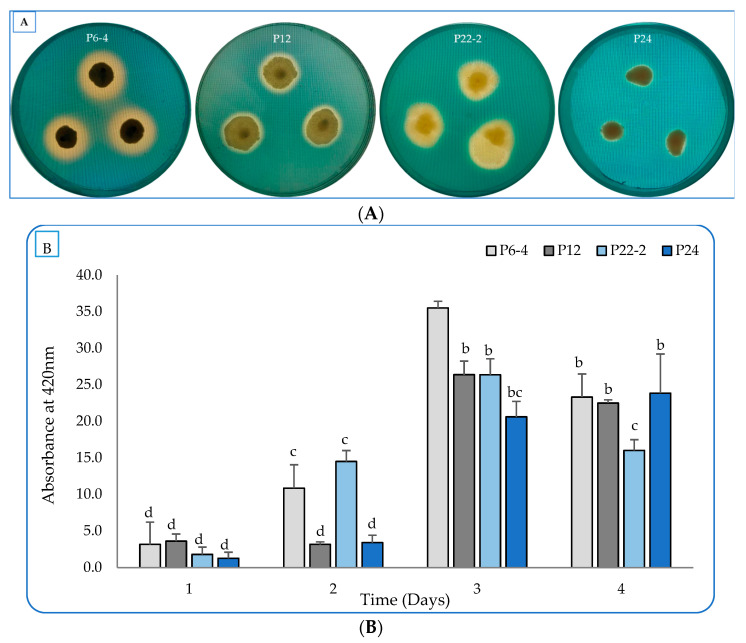
Detection of siderophore production by the four selected isolates (P6-4, P12, P22-2, and P24). (**A**) Growth of colonies on CAS-Nutrient agar medium with yellow–orange and clear halos indicating siderophore production. (**B**) The production efficiency in nutrient-broth medium for 4 days. All values represent the average of three replicates. Bars with different letters are significantly different (*p* > 0.05). Error bars represent the standard deviation of the mean.

Regarding the siderophore production, the four strains were tested on the blue CAS-Nutrient agar medium. The four strains displayed zones with varying colors, from yellow-orange to faint and clear around the growing colonies, indicating siderophore production (Figure 3A). When the production was quantified in the liquid cultures, the P6-4 strain produced the best amount of siderophore, reaching 35.51 psu, followed by the P12, P22-2, and P24 with 26.37, 26.36, and 23.84 psu, respectively (Figure 3B). The highest level of siderophore production was measured at day 3 for isolates P6-4, P12, and P22-2, whereas the P24 revealed the best amount after 4 days of incubation.

### 2.2. Detection of IAA Production in the Culture Broth by HPLC

**Figure 4:**
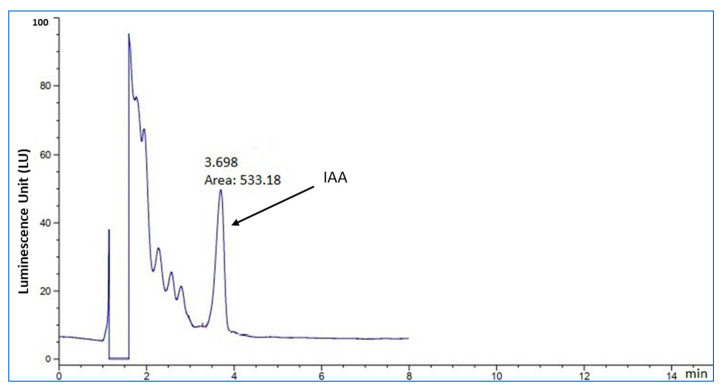
IAA detection by HPLC in the culture broth of P12 isolate grown for three days.

The extracted and purified broth of the growing P12 isolate was subjected to HPLC analysis. A significant peak after 3.689 min of retention was detected in the extracted broth, indicating IAA production (Figure 4). This peak was similar to the peak obtained from the synthetic IAA standard, which confirmed the production of the phytohormone by the selected isolate.

### 2.3. Identification of the Desired Plant-Growth-Promoting Strains

**Figure 5:**
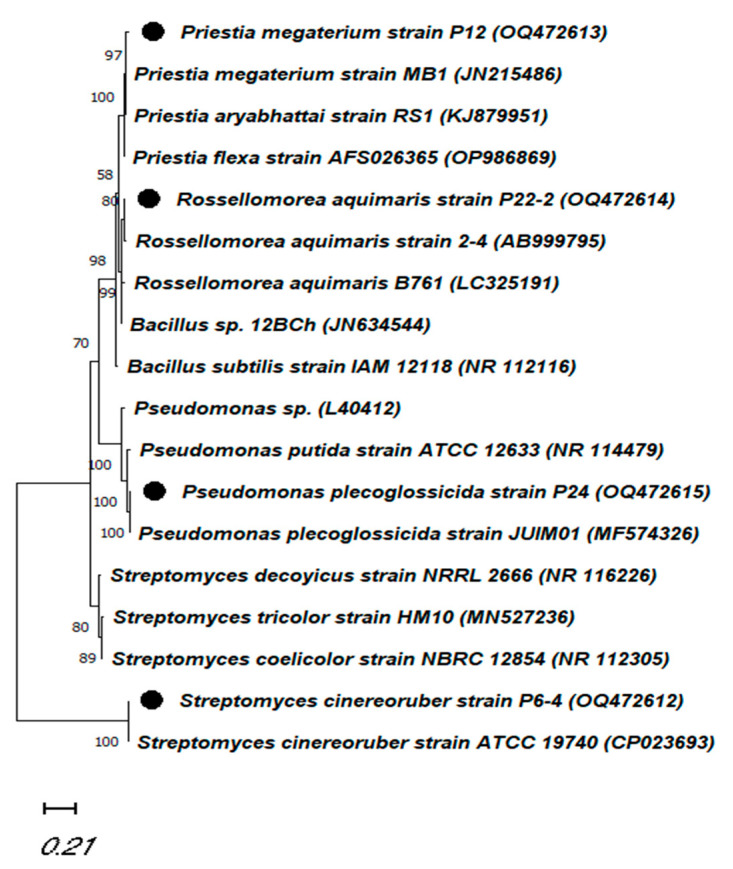
The constructed molecular phylogenetic tree inferred by using the maximum-likelihood method and Tamura-Nei model with 18 nucleotide sequences. ● points to our strains under study.

The products of the 16S rRNA fragment were sequenced to identify the promising isolates. The isolate P6-4 exhibited an identity with the *Streptomyces cinereoruber* strain ATCC 19740 (accession: CP023693) of 99.48%. In other cases, the two isolates P12 and P22-2 showed the highest degree of relatedness with the genus *Bacillus* (*Priestia*), and recorded similarity values of 99.72% and 98.55% with *Priestia megaterium* strain MB1 and *Rossellomorea aquimaris* (accessions: JN215486 and AB999795), respectively. The fourth isolate, P24, belonged to the genus *Pseudomonas*, with a level of homology with the *Pseudomonas plecoglossicida* strain, JUIM01 (accession: MF574326) that extended to 98.83%. The molecular-phylogenetic tree constructed using the neighbor-joining method revealed two groups (Figure 5). The first group contained four clades (one of which involved *Priestia megaterium* strain P12 and the second of which had *Rossellomorea aquimaris* P22-2, with *Pseudomonas plecoglossicida* P24 located in the third). Additionally, *Streptomyces cinereoruber* P6-4 was separated in the second group with *S. cinereoruber* ATCC 19740.

### 2.4. Assessing the Isolated PGP Bacteria on Tomato Plant

#### 2.4.1. Vegetative-Growth Traits

The desired strains under study were assessed for their potential ability to improve vegetative-growth traits, such as plant height, number of leaves per plant, root length, leaf dry matter (DM)%, stem DM%, and root DM%. The evaluated traits displayed a wide variation under the treatment conditions for the assessed traits at 21 and 45 DAT (Table 1). At 21 DAT, T1 (chemical fertilization with monophosphate, positive control), showed the best and most significant values for plant height (40.0 cm) and number of leaves per plant (9.67), whereas at 45 DAT, the significant and positive effects were assigned to T4 (rock phosphate + P12), with the plant height measured at 115.67 cm and the number of leaves per plant measured at 21.67. Furthermore, the T2 (RP) and T6 (RP + P24) recorded the lowest and most significant negative values for the same traits at 45 DAT. The same trend was observed in the root length and root dry matter (DM), since the T4 exhibited the most positive and significant root lengths, 15.0, and 26.67 cm, and root DM, measuring 12.6 and 12.48, at 21 and 45 DAT, respectively. On the other hand, no significant differentiations were detected in the stem or leaf dry-matter percentages, except the leaf DM in the T2 at 21 DAT. In general, the T4, followed by the T5 (RP + P22-2) were considered the most promising treatments related to plant height, number of leaves per plant, root length, and root DM.

The data related to the relative growth rate (mg g^−1^ day^−1^), net assimilation rate (mg cm^2^ d^−1^), and leaf area are presented in Table 2. The T4 treatment (RP + P12) and T5 (RP + P22-2) presented the highest and significant positive leaf area (770.39 and 6323.66 cm^2^) for this parameter at 21 and 45 DAT, respectively. Meanwhile, the T2 (RP) displayed the lowest values (398.45 and 3183.92 cm^2^) for the same trait with the same sequence, respectively. Furthermore, the net assimilation rate (NAR) after both dates manifested no significant differences between any of the treatments. Concerning the relative growth rate (RGR), positive and significant effects were obtained (0.15 mg g^−1^ d^−1^) by the T4 treatment after 21 DAT, whereas the best improvement after 45 DAT was recorded using the T5 treatment (0.14 mg g^−1^ d^−1^), without any significant effects.

#### 2.4.2. Soil and Plant Phosphorus

Due to the world’s limited P supplies, the utilization of plant-growth-promoting rhizobacteria will essentially replace phosphorus (P) fertilizers. The application PGPR enhances P solubilization and uptake by tomato plants. The obtained data in Table 3 demonstrate that the available soil phosphorus at 21 DAT was significantly higher with the T1 treatment (MSP) than with the other treatments, with improvements of up to 26.32% compared to the T2 (RP, negative control); this was followed by the T4 (RP + strain P12) treatment, which improved the P availability by 20.0% compared to the T2. At 45 DAT, the concentration of available soil phosphorus was significantly higher in both the T6 (RP + strain P24) treatment and the T1 (MSP), by 13.73% and 10.70%, respectively, followed by the T3 and T5, than in the RP, which gave the lowest P concentration in the soil.

Regarding the P uptake in the tomato plants, the data presented in Table 3 reveal that the most significant and positive leaf P uptake was detected in the T6 (RP + strain P24) and the T5 (RP + strain P22-2), with values of 123% and 112%, respectively, after 21 DAT, followed by the T4 (RP + strain P12), T1 (MSP), and T5 (RP + strain P22-2) with values of 51.9%, 41.0%, and 38.0%, respectively, after 45 DAT, compared with the T2. On the other hand, the T4 (RP + strain P12) caused a considerable increase in stem P uptake, with recorded percentages of 106.3 and 68.4 after 21 and 45 DAT, respectively. The same trend was observed in the root P uptake at 21 DAT, since the T4 showed the most significant improvement, of 87.1%, compared with the T2 (RP), whereas the most significant and positive P uptake in the roots after 45 DAT was presented by the T1, followed by the T5 (RP + strain P22-2). Eventually, the best total plant P-uptake values were demonstrated by the T6 and T5 (115.2% and 114.1%) at 21 DAT, whereas the T4 followed by T5, displayed the highest uptake, with 55.4% and 44.2%, respectively, at 45 DAT, without any significant differences.

### 2.5. Correlations between Studied Traits

**Figure 6:**
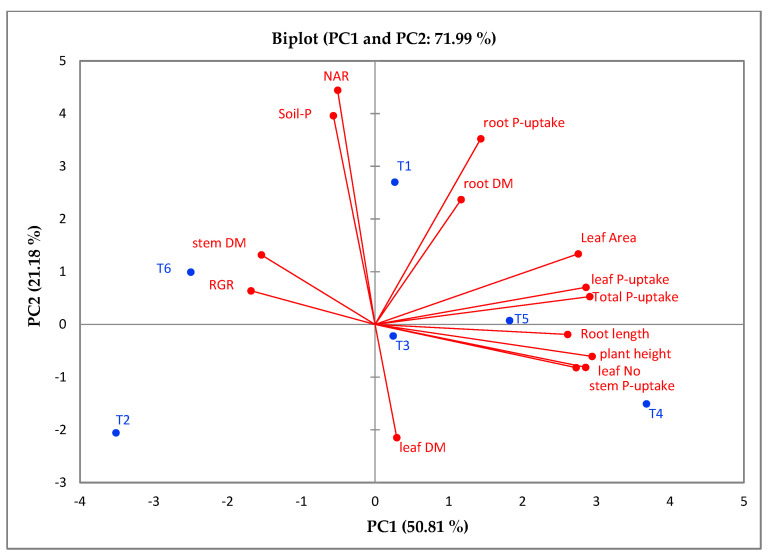
Biplot of PCAs describing the correlation between the evaluated traits in response to four treatments. PGPB in presence of either rock phosphate or mono-super phosphate.

The principal component analysis was used to assess the relationship between the estimated growth traits under the implemented treatments (PGPB in the presence of rock phosphate). The first two components of the PCAs measured 71.99% (PCA1 = 50.81% and PCA2 = 21.18%) at 45 DAT. Furthermore, these two PCAs were used to initiate the PC-biplot, as shown in Figure 6. A robust positive interrelation was detected between the plant height, leaf number, root length, root DM, leaf area, root P uptake, stem P uptake, leaf P uptake, and total P uptake. Furthermore, the treatments T1, T3, T4, and T5 were located on the positive side of the PCA1, in connection with most of the growth and phosphate-uptake traits, whereas the T2 (rock phosphate) appeared on the negative side of both the PCA1 and the PCA2, showing a negative correlation with all the evaluated traits.

**Figure 7:**
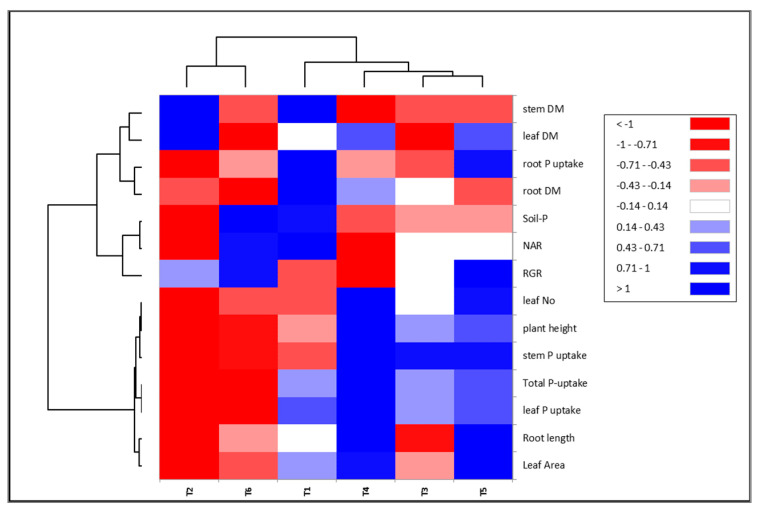
The heatmap correlation between the PGPB implemented with rock phosphate and the evaluated traits.

The heatmap analysis was employed to characterize the correlation between the applied treatments of PGPB and the evaluated vegetative and phosphate-uptake traits (Figure 7). The treatments were divided into two groups. One group included T1, T3, T4, and T5, while the second group contained T2 and T6. Accordingly, the traits under study were categorized into two groups. One group included the plant height, leaf number, leaf area, root length, stem P uptake, leaf P uptake and total P uptake, while the second group included the remaining traits (i.e., root DM, stem DM, leaf DM, NAR, RGR, root P uptake, and soil P). A significant and positive correlation was observed between the T4 and T5 treatments and traits such as the plant height, leaf number, leaf area, root length, stem P uptake, leaf P uptake and total P-uptake, whereas the T2 and T6 displayed a negative correlation with the same traits.

## 3. Discussion

In the rhizosphere, plant–microbe interactions affect plant health and productivity and soil fertility. Plant-growth-promoting bacteria (PGPB) are located in the rhizosphere and grow in, on, or around plant roots. They exert beneficial effects by enhancing plants’ growth, increasing their productivity, and protecting them from both diseases (i.e., soil-borne diseases) and abiotic stress [3,28]. Plant-growth-promoting bacteria stimulate plant growth via many mechanisms, including nitrogen fixation, the production of phytohormones (such as auxins), the solubilization of phosphate and potassium, siderophore production, 1-aminocyclopropane-1-carboxylate (ACC) deaminase activity, and the facilitation of the uptake of certain nutrients and water from the soil, increasing plants’ absorption of nutrients [3,6,28,29]. Numerous microorganisms, including *Streptomyces*, *Bacillus*, *Pseudomonas*, *Acinetobacter*, and *Rhizobium*, have the ability to increase plant growth. The isolation of new PGPB from the local environment is highly recommended to meet local environmental conditions. We isolated about 58 bacterial isolates on a NBRIP agar medium, and four isolates (P6-4, P12, P22-2, and P24) with phosphate-solubilization ability were chosen for further investigations. The four selected isolates were defined, based on the 16S rRNA, as *S. cinereoruber* strain P6-4 (OQ472612), *P. megaterium* strain P12 (OQ472613), *R. aquimaris* strain P22-2 (OQ472614), and *P. plecoglossicida* strain P24 (OQ472615).

Phosphate is considered a crucial element for various physiological and biochemical functions in plants. It is involved in the synthesis of nucleic acids (DNA and RNA), plays an essential role in the energy generation of ATP molecules, and enhances cell division and the development of new tissues in plants. Its production in crops and its quality traits are reduced under low P inputs in farm production [30]. Microorganisms dissolve P through the production of many compounds, such as exopolysaccharide, siderophores, organic acids, such as gluconic acid, which chelate the cations bound to phosphate, and inorganic acids (i.e., sulphuric, carbonic, and nitric acids). Furthermore, enzymes such as phosphatase, phytase, and C–P lyase are involved in organic-phosphate solubilization [31,32]. In our previous publication [14], the *S. thinghirensis* strain HM3 had a P solubilization efficacy that reached 84.4%, followed by the *S. tricolor* strain HM10 (81.6%). Yu et al. [33] isolated a highly efficient P-solubilizing bacterial strain, *Pseudomonas* sp. JP233, from soil; this strain can solubilize 258.07 mg/L in a NBRIP medium containing 5 g/L Ca_3_(PO_4_)_2_ in two days. Remarkably, four bacterial isolates, UC_1 (*Pseudomonas* sp.), UC_3 (*Chryseobacterium* sp.), UC_J (*Burkholderia* sp.), and UC_M (*Klebsiella* sp.), were applied to explore their effects on the growth of Chenmou elm (*Ulmus chenmoui*). These isolates showed high P solubilization with phosphate-solubility index (PSI) values 6.36, 1.71, 3.59, and 4.62, respectively [33].

The IAA is an essential molecule, which is secreted by rhizobacteria to stimulate and enhance plant growth and productivity. It regulates plant-growth and -development processes, such as the division, orientation, and elongation of cells, the differentiation of tissues, fertility, and apical dominance. Plant-growth-promoting bacteria secrete large amounts of IAA, which increases root development, enhancing the efficiency with which water and nutrients are obtained. Thus, plants improve their soil-volume exploitation, which leads to a reduction in fertilizer usage [34,35,36]. Notably, the P12 and P22-2 strains had gradual increases in IAA, displaying values of 251.70 and 236.57 µg/mL, respectively. Out of 30 rhizobacterial isolates, AB304, AB312, AB328, and AB331 synthesized up to 90 µg/mL or more of IAA, and the highest level of production was observed in the AB331, with 134.67  ±  3.59 µg/mL [37]. Lebrazi et al. [38] evaluated IAA production from eighty isolates of *Rhizobium* sp. isolate I69, with values of 135 μg/mL, followed by isolates I22 and I75, with efficacy values of 116 and 105 μg/mL, respectively. Furthermore, the inoculation of *Bacillus subtilis* and *Azospirillum brasilense* affected the growth of tomato plants through IAA synthesis. The *A. brasilense* produced IAA at a rate of 8.41 μg/mL in the supernatant, while the *B. subtilis* synthesized a considerable amount, reaching 76.34 μg/mL [39].

Siderophores are low-molecular-weight molecules secreted by bacteria to chelate iron from the surrounding environment. These molecules bind iron (Fe^3+^) with high affinity and help in its solubilization, which improvs growth and yield [40]. Iron plays important roles in photosynthesis and respiration process via the electron-transport chain. Furthermore, it helps to move oxygen throughout plants’ parts, producing their green color, and it is a co-factor in many vital enzymes [41]. The *Streptomyces tricolor* strain HM10 secreted up to 64.14% psu from siderophores after 4 days of incubation [14,15,42]. Cherif-Silin et al. [43] isolated thirty-five *Bacillus* strains from a wheat rhizosphere. Three strains (B25, D11, and BA11) were able to produce siderophores. Additionally, *Bacillus* spp. secretes several substances that improve plant growth and control pathogen infection. In addition, *Bacillus* produces exopolysaccharides and siderophores under conditions of water scarcity and salinity as an adaptation to abiotic stress [44,45].

When assessing the growth and P-uptake traits in the tomato plants in the presence of PGPR, the mono super-phosphate (MSP) and rock phosphate (RP) displayed significant and positive effects. Accordingly, the T4 (RP + P12), followed by the T5 (RP + P22-2) were considered the most promising treatments related to plant height, number of leaves per plant, root length, leaf area, leaf P uptake, stem P uptake, and total plant P uptake. These findings are in accordance with those in a previously published reports by Singh et al. [46], which found a significant improvement in root and shoot length, fresh and dry weights of roots and shoots, and lateral-root number in mung bean (*Vigna radiata*) by *Burkholderia arboris* (isolate CSRS12). Furthermore, *Bacillus mojavensis* increased the yield of sweetcorn by 16%, while *B. subtilis* yielded an increase of 13.8%, and *B. pumilus* produced an increase of 11.8%, with higher protein and fiber contents in the grains to the control [4]. Phares et al. [47] and Kaur et al. [48] stated that phosphate-solubilizing bacteria (PSB) are important components of sustainable agriculture, since PSB increased the availability of phosphate by mineralizing the insoluble fraction of inorganic phosphate. The authors noted considerable improvements in the plant-growth parameters, phosphorus (P) content, and soil availability of P with bacterial treatments using *Bacillus* spp., *Pseudomonas* spp., and *Arthrobacter* spp., in potatoes and maize.

The application of the relationship between the evaluated traits and PGPR treatments could reinforce the efficiency of PGPR in sustainable agriculture. To discover the link between the traits under study, the PC biplot is a suitable method. A powerful positive interrelation between plant height, leaf number, root length, root DM, leaf area, root P uptake, stem P uptake, leaf P uptake, and total P uptake was observed. Furthermore, the heatmap analysis demonstrated the correlation between the applied treatments and the evaluated vegetative and phosphate-uptake traits. The traits and treatments under study were categorized in two groups. The assessment of 127 diverse bread-wheat genotypes for their drought tolerance demonstrated that PC1, PC2, and PC3 accounted 35.4%, 24.6%, and 11.6% of the total variation; furthermore, the first two components of the PCAs represented 55.83% and 22.80%, respectively, of the variability in the wheat’s tolerance of heat [49,50]. Additionally, the evaluation of the salt tolerance of 25 tomatoes (*S. lycopersicum* L.) disclosed that the PC1 measured 24.3% of the variability, followed by the PC2, with 16.2%). Furthermore, the PCA-biplot and cluster-heatmap analyses indicated that some of the tomato accessions were salt-tolerant (i.e., Subarctic, Raad-Red, Naqeeb, Pakit, Tommy-Toe, and BL-1076) [49]. The combination of cyanobacteria with the promising cytoplasmic male sterility (CMS) lines is considered a useful tool for enhancing the outcrossing rates and improving the seed production of hybrid rice [51].

## 4. Materials and Methods

### 4.1. Bacterial Isolation and Growth Conditions

Soil samples collected from Qassim region (from the rhizosphere area of health plants), Saudi Arabia, were subjected to bacterial isolation by serial-dilution method on National Botanical Research Institute’s phosphate (NBRIP) agar medium, composed of glucose (10 g), Ca_3_(PO_4_)_2_ (5 g), MgCl_2_.6H_2_O (5 g), MgSO_4_.7H_2_O (0.25 g), (NH_4_)_2_SO_4_ (0.1 g), KCl (0.2 g), agar (15 g), and dH_2_O up to 1 L [52]. Bacteria with clear zones around colonies (58 isolates), indicating phosphate solubilization, were selected, isolated, and maintained. Four strains were chosen, based on the efficiency with which they solubilized phosphate, for further investigation, and their growth and maintenance were performed on nutrient broth/agar medium for 24–48 h at 30 °C.

### 4.2. Phosphate Solubilization, IAA, and Siderophore Production of Selected Strains

Four isolates, designated P6-4, P12, P22-2, and P24, with phosphate-solubilization activities were selected to evaluate their plant-growth-promoting activity. For detecting phosphate solubilization, pure culture from each strain was grown on/in NBRIP agar/broth medium for 4 days in incubator shaker at 30 °C and 200 rpm. Plates with clear halos around growing colonies were marked as positive in phosphate solubilization. To measure P-solubilization efficiency in liquid medium, samples at were collected at days 0, 1, 2, 3, and 4 from inoculated-culture broth and subjected directly to measurement of absorbance at 420 nm in microplate reader (EPOCH2TS, BioTek, Winooski, VT, USA). The solubilization activity was calculated as a decrease in absorbance in comparison to uninoculated medium. The IAA and IAA-like-molecule production was evaluated in cultures grown in nutrient broth (NB) medium supplemented with 0.2% L-tryptophan under 200 rpm of shaking and 30 °C incubation for 4 days. Every 24 h, one milliliter from each culture was withdrawn and stored at −20 °C. Based on the method described by Gang et al. [53], 100 µL of Salkowaski reagent (FeCl_3_, 0.5 M; HClO_4_, 35%) was mixed with an equal volume (100 µL) of the culture supernatant (centrifuged at 10,000 rpm for 5 min) in a 96-well microplate and incubated in dark for 30 min at room temperature. After color development, the mixture was spectrophotometrically measured at 535 nm in presence of both standard curve generated by known concentrations from IAA and uninoculated NB medium.

Applying siderophore detection and quantification, CAS assay (Chrome Azurol S) [54,55] was used by preparing CAS solution (60.5 mg of chrome azurol S in 50 mL dH_2_O + 72.9 mg of hexadecyl trimethyl ammonium bromide in 40 mL ddH_2_O + 10 mL Fe^3+^ (1 mM FeCl_3_ in 10 mM HCl)) and combined with nutrient agar (NA) with a 1:9 ratio for siderophore detection. Furthermore, CAS solution was mixed with culture supernatant (centrifuged at 10,000 rpm for 5 min) by mixing equal volumes (100 µL) of each. The combined mixture was incubated at room temperature for 20 min and the absorbed wavelength was measured at 630 nm by the microplate reader [56]. The percentage siderophore unit (psu) was calculated by the method described by Payne [57], according to the following formula:

$$\mathrm{percent}\;\mathrm{siderophore}\;\mathrm{unit}\;\left(\mathrm{psu}\right)=\frac{\left(\mathrm{Ar}-\mathrm{As}\right)\times 100}{\mathrm{Ar}}$$

where Ar = absorbance of medium not inoculated with CAS, As = absorbance supernatant in cultured medium with CAS.

### 4.3. IAA Detection in the Culture Broth by HPLC

The fermented culture broth of P12 strain grown in NB in presence of 0.2% L-tryptophan for 72 h was centrifugated at 12,000 rpm for 5 min. A total of 10 mL from the filtrate was mixed with equal volume of acidified acetonitrile (0.1% acetic acid) with 2 min shaking. Next, 1 g of sodium acetate and 4 g of MgSO_4_ were added with shaking for 1 min, followed by centrifugation at 10,000 rpm for 5 min. The mixture was filtrated through a 0.45-µm-syringe filter and diluted with 2 volumes of water for HPLC-FLD analysis according to the following measurements: 1220 infinity HPLC (Agilent, USA), Column (C18-E, 4.6 × 100 mm, 5-µm particle size, VD sphere PUR 100 -Agilent), fluorescence light detector (FLD) (excitation 282 nm, emission 360 nm), and mobile phase of 0.8 mL/min. Gradient elution comprised: (A) water (PH 3.8); (B) acetonitrile, 0 min (A, 80%:B, 20%), 25 min (A, 50%:B, 50%), and 31 min (A, 80%:B, 20%), with column temperature of 30 °C and sample injection of 20 µL.

### 4.4. Bacterial Identification by 16S rRNA, Accession Numbers, and Phylogenetic-Tree Construction

The DNA was extracted from cultures grown in NB for 24 h according to the method presented by Cook and Meyers [58]. The 16S-rRNA region was amplified with the general primers 27F, 5′ AGAGTTTGATCATGGCTCAG 3′ -and 1492R, 5′ TACGGTTACCTTGTTACGACTT 3′. The amplified products were electrophoresed in 1% agarose gel for amplification detection, sequenced (Macrogen company, Seoul, South Korea), assembled (CLC Main Workbench 8), and submitted to NCBI for accession numbers. The molecular-phylogenetic tree was constructed by MEGA 11 [59] with the following parameters: maximum likelihood, bootstrap method (1000 replication), and Tamura-Nei model with nearest-neighbor interchange (NNI). The 16S-rRNA sequences were deposited in GenBank under the following accession numbers: OQ472612–OQ472615.

### 4.5. Greenhouse Experiment

#### 4.5.1. Experimental Site and Agriculture Conditions

The current experiment was performed at the experimental demonstration farm, College of Agriculture and Veterinary Medicine, Qassim University, Saudi Arabia (longitude 44–45 E, latitude 26–27 N, altitude 725 m above sea level). The influence of four isolated PGPR as biofertilizers in the presence of rock phosphate on the vegetative growth of tomato plants and phosphate solubilization was investigated. The characteristics of soil samples and other details are shown in Table 4.

The use of NPK as mineral fertilizer, recommended for tomato plants, was implemented. The doses were as follows: 285 kg N/ha as 620 kg urea (46% N), 390 kg calcium superphosphate (18.5% P_2_O_5_) or 72 kg P_2_O_5_/ha as 248 kg rock phosphate (29% P_2_O_5_) as a control and 238 kg K_2_O/ha as 661 kg potassium thiosulphate (36% K20). The total amounts of calcium superphosphate and rock phosphate were added to pots once before transplantation. Urea and potassium thiosulphate were added every week, from week of transplantation to the end of experiment. Micronutrients (Fe, Zn, Mn, Cu, and Mo) were added three times, as recommended, in form of leaf spray.

#### 4.5.2. PGPB Treatments under Greenhouse Conditions

The four selected bacterial strains were grown in NB medium for 48 h with shaking at 200 rpm and at 30 °C. The broth cultures (with optical density adjusted at 600 nm to approximately OD_600_ = 0.8) were prepared for inoculation by adding 5 g/100 mL of glucose as a primary carbon source. After transplanting the tomato plants, each isolate was used to inoculate seedlings by adding the culture broth (nearly 40 mL per plant) close to roots. The inoculation process was performed twice at the time of transplantation and after two weeks to confirm the presence of bacteria around plant roots.

Six fertilization treatments involving mono super-phosphate (MSP, T1) as positive control, rock phosphate (RP, T2) as a negative control, RP + strain P6-4 (T3), RP + strain P12 (T4), RP + strain P22-2 (T5), and RP + P24 (T6) were implemented. Rock-phosphate and mono super-phosphate treatments were added to soil in each pot (30 cm in length by 30 cm in diameter, filled with 20.7 kg of autoclaved sterilized sandy soil at 121 ℃ for 30 min) before transplantation at a rate of 4.8 g of MSP and 7.5 g of RP.

The seedlings of Marlen F.1 tomato hybrid cultivar were purchased from the Saudi United Fertilizers Company. Next, the seedlings were transferred into pots inside the greenhouse in a complete randomized block design with three replicates, and each treatment involved twelve plants. The average temperatures at day and night in greenhouses were 25 °C and 18 °C, respectively, in line with optimum temperatures for tomato growth described by Maynard and Hochmuth [63].

#### 4.5.3. Data Collected

##### The Growth Traits

After 21 DAT and 45 DAT, three plants from each treatment were randomly selected. Digging was performed around plants and the whole plants, including roots and soil around roots, were collected to measure the following characteristics: plant height (cm), number of leaves per plant, root length (cm), root-dry-matter percentage (root DM), stem-dry-matter percentage (stem DM), and leaf-dry-matter percentage (leaf DM), after 72 h of drying at 70 °C. Leaf area (LA, cm^2^) was calculated according to Schwarz and Klaring [64] and Blanco and Folegatti [65].

Furthermore, the relative growth rate (RGR) (mg g^−1^ d^−1^) was estimated based on Hunt et al. [20] and Evans [66] by applying the following equation:

$$\mathrm{RGR}\;=\frac{{\mathrm{Ln}\;W}_{2}-{\;\mathrm{Ln}\;W}_{1}}{T_{2}-{\;T}_{1}}\left(\mathrm{mg}\;{\mathrm{cm}}^{-2}\;d^{-1}\right)$$

where Ln is logarithm of the natural base, W_1_ is dry weight of the plant at the beginning of the period T_1_, W_2_ is dry weight of the plant at the end of the period T_2_, and W_1_ and W_2_ are the total DW (mg/plant).

On the other hand, net assimilation rate (NAR) (mg cm^−2^ d^−1^) was measured by the following equation, in accordance with Evans [66] and Hunt et al. [20].

$$\mathrm{NAR}\;=\frac{W_{2}-W_{1}}{T_{2}-{\;T}_{1}}\times \frac{{\mathrm{Ln}\;\mathrm{LA}}_{2}-\mathrm{Ln}\;\mathrm{LA}}{{\mathrm{LA}}_{2}-{\;\mathrm{LA}}_{1}}\left(\mathrm{mg}\;{\mathrm{cm}}^{-2}\;d^{-1}\right)$$

where W_1_ is dry weight of the plant at the beginning of the period T_1_, W_2_ is dry weight of the plant at the beginning of the period T_2_, LA_1_ is leaf area of the plant at the beginning of the period T_1_, Ln is logarithm of the natural base and LA_2_ is leaf area of the plant at the beginning of the period T_2_.

Root, stem, and leaf DMs were assessed after the two aforementioned periods (21 and 45 DAT).

##### Phosphorus Analysis

Three rhizospheric soil samples were collected randomly from each treatment at 21 and 45 DAT for measuring available phosphorus. Soil P was extracted using 1:20 of soil and 0.5M NaHCO3 at pH 8.5. Available soil P concentrations were determined using ascorbic-acid colorimetric method with microplate reader (EPOCH2TS) spectrophotometer at 880 nm [67].

At the same two DATs, three plants were randomly collected and oven-dried at 70 °C for 72 h, and ground to pass through a 0.5-mm sieve. Levels of plant phosphorus were measured in dry matter of plant leaves, stems, and roots. The samples were wet-digested using concentrated H_2_SO_4_ and H_2_O_2_. The P concentrations were measured calorimetrically by ascorbic-acid colorimetric method using microplate-reader spectrophotometer according to Cottenie et al. [68]. The uptake of P by leaves, stems, and roots was calculated by multiplying leaf, stem, and root DM (g plant^−1^) by the respective value of P concentration for a particular treatment. The total P uptake (expressed as mg plant^−1^) in the individual treatment was calculated from the total P uptake by leaves, stems, and roots.

##### Statistical Analysis and Principal Component Analysis

Collected data were statistically analyzed, and Duncan’s test was applied for comparison of means at the significant level (*p* ≤ 0.05), as illustrated by Khiddir et al. [69] and Webster [70]. The correlation between different variables was measured by Pearson correlation and the normality of variables was verified using the Shapiro–Wilk test before proceeding with principal component analysis (PCA). Furthermore, Bartlett’s sphericity and Kaiser–Meyer–Olkin (KMO) tests were applied. The PCA and heatmap were created using XLSTAT software version 2019 [71].

## 5. Conclusions

Plant-growth-promoting bacteria enhance plant growth by producing a variety of metabolites, such as IAA, siderophores, and ACC deaminase, as well as phosphate solubilization, and nitrogen fixation. The four isolated strains exhibited a pattern of improvement in tomato-plant growth and P uptake by inducing IAA and siderophore production, in addition to P solubilization.

The *Priestia megaterium* strain P12 (T4), followed by the *Rossellomorea aquimaris* strain P22-2 (T5), showed the best values for growth and P-uptake-related traits, such as plant height, number of leaves per plant, root length, leaf area, leaf P uptake, stem P uptake, and total plant P uptake. It can be concluded that IAA production and P-solubilization efficacy play major roles in plant-growth enhancement.

## Figures and Tables

**Table 1:** Effects of chemical (mono-super phosphate), organic (rock phosphate), and PGPB (biofertilizer) treatments on some vegetative-growth traits (plant height (cm), no. of leaves plant^−1^, root length (cm), leaf DM%, stem DM%, and root DM%) of tomato plants grown under greenhouse conditions 21 and 45 days after transplantation (DAT).

Treatments	Plant Height (cm)		No. of Leaves Plant^−1^		Root Length (cm)		Leaf DM (%)		Stem DM (%)		Root DM (%)	
	21DAT	45DAT	21DAT	45DAT	21DAT	45DAT	21DAT	45DAT	21DAT	45DAT	21DAT	45DAT
T1	40.00 ^a^	95.00 ^bc^	9.67 ^a^	15.67 ^bc^	12.67 ^ab^	21.33 ^ab^	8.27 ^b^	12.22 ^a^	4.84 ^a^	8.23 ^a^	9.54 ^b^	14.20 ^a^
T2	33.67 ^ab^	82.33 ^d^	9.33 ^a^	13.33 ^c^	14.27 ^ab^	18.00 ^b^	9.86 ^a^	12.82 ^a^	4.49 ^a^	8.58 ^a^	9.82 ^b^	11.60 ^a^
T3	35.33 ^ab^	101.00 ^b^	8.67 ^ab^	17.33 ^bc^	10.87 ^b^	19.00 ^ab^	8.10 ^b^	11.57 ^a^	4.69 ^a^	7.12 ^a^	8.94 ^b^	12.09 ^a^
T4	34.67 ^ab^	115.67 ^a^	8.67 ^ab^	21.67 ^a^	15.00 ^a^	26.67 ^a^	8.38 ^b^	12.41 ^a^	5.15 ^a^	6.77 ^a^	12.60 ^a^	12.48 ^a^
T5	31.50 ^ab^	102.67 ^b^	8.00 ^b^	19.33 ^ab^	14.50 ^ab^	25.33 ^ab^	8.05 ^b^	12.48 ^a^	4.60 ^a^	7.08 ^a^	10.44 ^b^	11.65 ^a^
T6	30.00 ^b^	87.50 ^cd^	7.67 ^b^	15.50 ^bc^	11.97 ^ab^	20.50 ^ab^	8.16 ^b^	11.52 ^a^	4.72 ^a^	7.03 ^a^	10.24 ^b^	10.93 ^a^

Mono-super phosphate (MSP, T1), rock phosphate (RP, T2), RP + strain P6-4 (T3), RP + strain P12 (T4), RP + strain P22-2 (T5) and RP + strain P24 (T6), dry matter (DM), days after transplantation (DAT). Values followed by different letter(s) within each column significantly differ according to the Duncan multiple-comparison test at the 5% level. Each value is the average of 3 replicates.

**Table 2:** Effects of chemical (mono-super phosphate), organic (rock phosphate), and PGPB (biofertilizer) treatments on some vegetative-growth traits (leaf area (cm^2^), net assimilation rate (mg cm^−2^ day^−1^), relative growth rate (mg g^−1^ day^−1^)) of tomato plants grown under greenhouse conditions 21 and 45 days after transplantation (DAT).

Treatments	Leaf Area (LA) (cm^2^)		Net Assimilation Rate (NAR) (mg cm^2^ d^−1^)		Relative Growth Rate (RGR) (mg g^−1^ d^−1^)	
	21DAT	45DAT	21DAT	45DAT	21DAT	45DAT
T1	532.71 ^ab^	5392.97 ^ab^	0.46 ^a^	0.48 ^a^	0.12 ^b^	0.12 ^a^
T2	398.45 ^b^	3183.92 ^c^	0.35 ^a^	0.35 ^a^	0.12 ^b^	0.13 ^a^
T3	556.18 ^ab^	4799.84 ^abc^	0.43 ^a^	0.42 ^a^	0.13 ^ab^	0.13 ^a^
T4	770.39 ^a^	5893.34 ^ab^	0.47 ^a^	0.34 ^a^	0.15 ^a^	0.11 ^a^
T5	550.55 ^ab^	6323.66 ^a^	0.47 ^a^	0.42 ^a^	0.14 ^ab^	0.14 ^a^
T6	473.34 ^b^	4247.94 ^bc^	0.48 ^a^	0.47 ^a^	0.13 ^ab^	0.13 ^a^

Mono-super phosphate (T1), rock phosphate (RP, T2), RP + strain P6-4 (T3), RP + strain P12 (T4), RP + strain P22-2 (T5), and RP + strain P24 (T6), days after transplantation (DAT). Values followed by different letter(s) within each column significantly differ according to the Duncan multiple-comparison test at the 5% level. Each value is the average of 3 replicates.

**Table 3:** Effects of chemical (mono-super phosphate), organic (rock phosphate), and PGPB (biofertilizer) treatments on soil- and plant-phosphorus concentrations.

Treatment	Plant P-Uptake (mg Plant^−1^)								Soil-P conc. (mg kg^−1^)	
	Leaf		Stem		Root		Total			
	21 DAT	45 DAT	21 DAT	45 DAT	21 DAT	45 DAT	21 DAT	45 DAT	21 DAT	45 DAT
T1	8.42 ^b^	98.04 ^a^	1.71 ^ab^	26.89 ^abc^	0.39 ^ab^	3.35 ^a^	10.52 ^b^	130.89 ^ab^	12.00 ^a^	24.10 ^a^
T2	8.71 ^b^	69.52 ^b^	1.27 ^b^	22.27 ^c^	0.31 ^b^	1.59 ^c^	10.29 ^b^	93.38 ^c^	9.50 ^d^	21.77 ^c^
T3	17.09 ^ab^	92.76 ^ab^	1.79 ^ab^	35.33 ^ab^	0.46 ^ab^	1.95 ^c^	19.34 ^ab^	130.04 ^ab^	10.70 ^c^	22.78 ^b^
T4	12.46 ^ab^	105.61 ^a^	2.62 ^a^	37.50 ^a^	0.58 ^a^	2.24 ^bc^	15.66 ^ab^	145.35 ^a^	11.40 ^b^	22.35 ^bc^
T5	18.54 ^a^	95.94 ^a^	2.21 ^ab^	35.77 ^ab^	0.28 ^b^	2.96 ^ab^	21.03 ^a^	134.67 ^a^	10.70 ^c^	22.67 ^b^
T6	19.46 ^a^	69.67 ^b^	2.29 ^ab^	24.57 ^bc^	0.38 ^ab^	2.09 ^bc^	22.14 ^a^	96.11 ^bc^	10.60 ^c^	24.76 ^a^

Mono-super phosphate (MSP, T1), rock phosphate (RP, T2), RP + strain P6-4 (T3), RP + strain P12 (T4), RP + strain P22-2 (T5), and RP + strain P24 (T6), days after transplantation (DAT). Values followed by the same letter(s) within each column did not significantly differ according to the Duncan multiple-comparison test at the 5% level. Each value is the average of 3 replicates.

**Table 4:** Physical and chemical properties of soil and water used for the study.

Properties	Value	
	Soil	Water
Physical properties		
Sand (%)	93.4	-
Silt (%)	3.9	-
Clay (%)	2.7	-
Texture	Sand	-
Chemical properties		
^1^ pH	7.82	7.27
^2^ EC (dS m^−1^)	1.04	0.95
^3^ Nutrients (ppm)		
Total N	175	-
Available P	1.26	-
Available K	74.0	42.0
^4^ Dissolved ions (meq L^−1^)		
Dissolved anions (meq L^−1^)		
Cl^−^	8.7	8.2
HCO_3_^−1^ + CO_3_^−2^	2.1	1.7
Dissolved cations (meq L^−1^)		
Na^+^	7.3	7.1
Ca^++^	2.8	1.8
Mg^++^	0.9	0.9

In accordance with Page [60], ^1^ pH was measured in soil suspension at a ratio of (1:2.5) using a pH meter (Jenway, model 3310). ^3^ Total N was determined by Kjeldahl method; P was extracted and measured using Olsen method; K by 1 N NH_4_OAc at pH 7. ^2^ EC (dSm^−1^) was determined in the saturated soil-paste extract using an EC meter (ELE, model 470), as recommended by Jackson [61]. ^4^ The soluble cations and anions were measured in saturated soil-paste extract by titration method using EDTA solution for calcium and magnesium, while carbonate and bicarbonate were measured by titration with hydrochloric acid, and chloride was measured by titration with silver nitrate, whereas sodium and potassium were determined using a flame photometer, according to the method mentioned by Jackson [61]. Particle-size distribution of the soil samples was determined using hydrometer method [62].

## Data Availability

Data are available upon request.
